# *Bacillus subtilis* vector based oral rabies vaccines induced potent immune response and protective efficacy in mice

**DOI:** 10.3389/fmicb.2023.1126533

**Published:** 2023-02-09

**Authors:** Ying Zhang, Ruo Mo, Sheng Sun, Zhanding Cui, Bo Liang, Entao Li, Tiecheng Wang, Ye Feng, Songtao Yang, Feihu Yan, Yongkun Zhao, Xianzhu Xia

**Affiliations:** ^1^Northeast Forestry University College of Wildlife and Protected Area, Harbin, China; ^2^Changchun Veterinary Research Institute, Chinese Academy of Agricultural Sciences, Changchun, Jilin, China; ^3^College of Veterinary Medicine, Jilin Agricultural University, Changchun, Jilin, China; ^4^State Key Laboratory of Veterinary Etiological Biology, Lanzhou Veterinary Research Institute, Chinese Academy of Agricultural Sciences, Lanzhou, Gansu, China; ^5^Division of Life Sciences and Medicine, University of Science and Technology of China, Hefei, Anhui, China

**Keywords:** rabies, oral immunization, *Bacillus subtilis*, oral rabies vaccine, G protein

## Abstract

**Introduction:**

Rabies is a worldwide epidemic that poses a serious threat to global public health. At present, rabies in domestic dogs, cats, and some pets can be effectively prevented and controlled by intramuscular injection of rabies vaccine. But for some inaccessible animals, especially stray dogs, and wild animals, it is difficult to prevent with intramuscular injection. Therefore, it is necessary to develop a safe and effective oral rabies vaccine.

**Methods:**

We constructed recombinant *Bacillus subtilis* (*B. subtilis*) expressing two different strains of rabies virus G protein, named CotG-E-G and CotG-C-G, immunogenicity was studied in mice.

**Results:**

The results showed that CotG-E-G and CotG-C-G could significantly increase the specific SIgA titers in feces, serum IgG titers, and neutralizing antibodies. ELISpot experiments showed that CotG-E-G and CotG-C-G could also induce Th1 and Th2 to mediate the secretion of immune-related IFN-γ and IL-4. Collectively, our results suggested that recombinant *B. subtilis* CotG-E-G and CotG-C-G have excellent immunogenicity and are expected to be novel oral vaccine candidates for the prevention and control of wild animal rabies.

## Introduction

Rabies is a natural zoonotic infectious disease, which is prevalent worldwide and poses a serious threat to global public health (Taylor and Nel, 2015). The disease is prevalent all over the world and poses a serious threat to global public health. Once clinical symptoms appear after infection, the mortality rate is almost 100% (Henle et al., 1962; Fenje, 1968). About 60,000 people die of the disease every year (Yousaf et al., 2012; Kip et al., 2018), and 95% of the cases are from developing countries such as Africa and Asia (Luo et al., 2017). Dogs, especially stray dogs, are the main source of rabies transmission in developing countries (Hampson et al., 2015; Zhao et al., 2021). In developed countries, bats and other wild animals are the main culprits in the spread of rabies. In recent years, the study of rabies in wild animals has been reported all over the world. Wild animals are the main reservoir of the Rabies virus (RABV) in nature. Human and domestic rabies deaths are frequently caused by wild animal bites (Shao et al., 2015; Ming-Yang et al., 2018; Benavides et al., 2020; Feng et al., 2022). It brings serious mental pressure and economic loss to people. Therefore, enhanced rabies vaccination of stray dogs and wild animals is essential for global rabies control, and the most practical way to vaccinate these animals against rabies is the oral vaccine. Currently, the widely used Oral rabies vaccine (ORV) is mainly divided into the live attenuated vaccine and recombinant live rabies virus vector vaccine. Most notable are the poxvirus-vectored rabies glycoprotein recombinant virus vaccines, such as recombinant vaccinia virus and canary poxvirus expressing the rabies virus glycoprotein gene. Both vaccines are widely used for oral rabies vaccination in wild animals in North America, Canada, and Western Europe (Poulet et al., 2007; Weyer et al., 2009). At present, with the in-depth study of epidemiology, rabies in pet dogs, cats, and some domestic animals has been effectively prevented and controlled by an intramuscular rabies vaccine, but it is not suitable to prevent rabies in wild animals. Therefore, enhanced rabies vaccination of stray dogs and wild animals is essential for global rabies control, and the most practical way to vaccinate these animals against rabies is the oral vaccine. So, the development of a simple, safe and effective oral rabies vaccine for wild animals is of great significance for the prevention and control of animal rabies.

RABV, a typical neurotropic virus, is a prototypical virus in the genus *Lyssavirus* genus, family Rhabdoviridae (Wunner, 2013; Liu et al., 2017). The RABV genome is about 12 kb in length and consists of five genes encoding nucleocapsid protein (N), phosphoprotein (P), matrix protein (M), glycoprotein (G), and viral RNA polymerase (L) (Conzelmann et al., 1990). Among them, G protein is the only surface-exposed viral protein on RABV virions, and it is the only virus protein that stimulates virus neutralizing antibodies (Wiktor et al., 1973). The G protein is an important determinant for the induction of innate immune responses and of cellular and humoral immune responses required to confer complete protection in animals (Cox et al., 1977; Benjamin et al., 2015) therefore, it is a promising candidate antigen for the development of genetically engineered vaccines.

*Bacillus subtilis* is a Gram-positive bacterium that is safe and non-toxic to humans and animals. It has been certified as a Generally Regarded as Safe (GRAS) product and is a new type of probiotic currently widely used (Hong et al., 2005; Schumann, 2007; Elshaghabee et al., 2017). Spores have unique stress resistance and can survive high temperature, dry and acid–base invasion conditions. *B. subtilis* and its spores are widely used as antigenic protein delivery vehicles and mucosal immune enhancers and can cause a protective immune response. At present, the exogenous protein system displayed on the surface of *B. subtilis* spores is more mature and perfect, and the recombinant expression of exogenous proteins will not affect the structure of *B. subtilis* spores and the performance of survival in harsh environments. Antigens displayed on the surface of spores can also be used for immunization and can germinate in host phagocytes, resulting in an efficient antigen presentation process. Currently, in the research of vaccine against tetanus toxin and *Bacillus anthracis* protective antigens (Duc et al., 2007; Sangun et al., 2010), the spore-presenting candidate vaccine with *B. subtilis* spore as antigen display carrier has shown good immunogenicity and immune protection. In recent years, a variety of spore surface proteins, such as CotB, CotC, and CotG (Mauriello et al., 2004; Imamura et al., 2011; Mou et al., 2016), have been widely used in the system of displaying foreign proteins on the spore surface.

In this study, the results showed that both recombinant *B. subtilis* were successfully expressed. Both strains of recombinant *B. subtilis* can induce mice to produce higher levels of specific antibodies after oral immunization and improve the production and protective effect of VNA, suggesting that recombinant *B. subtilis* is a novel oral rabies vaccine candidate.

## Materials and methods

### Ethics statement

All BALB/c mice were purchased from Beijing Weitong Lihua Experimental Animal Technology Co., Ltd., and the animal experiments were approved by the Animal Welfare and Ethics Committee of Changchun Veterinary Research Institute under the license number JSY-DW-2018-02. All BALB/c mice were treated according to the Guidelines for the Welfare and Ethics of Laboratory Animals of China (GB 14925–2001). Experiments related to virulent strains of RABV were all carried out in the biosafety level III laboratory.

### Bacterial strains, cells, viruses, and antibodies

*Bacillus subtilis* 168 strain (BS168) was purchased from Wuhan Pujian Biotechnology Co., Ltd. PDG1661 plasmid was purchased from BioVector Plasmid Vector Strain Cell Gene Collection Center. HuNPB3 street strain, BHK cells, and NA cells were stored in our laboratory. Reference serum from the rabies reference Laboratory of the World Organization for Animal Health. A fluorescein isothiocyanate (FITC)-conjugated monoclonal antibody (mAb) against RABV N protein was purchased from Fujirebio, an anti-RABV G protein mAb was purchased from Millipore, Horseradish peroxidase (HRP)-conjugated goat anti-mouse IgG was purchased from Abcam. HRP-conjugated goat anti-mouse IgG1, IgG2a, and IgA were purchased from Southern Biotech Company. IFN-γ and IL-4 ELISpot plates were purchased from MABTECH Company. Chemiluminescent Imaging System were purchased from Tanon Company.

### Construction of recombinant *Bacillus subtilis*

Using the codon-optimized pUC57-CotG-E-G and pUC57-CotG-C-G (E-G, GenBank: J02293; C-G, GenBank: GQ918139) as templates, design primers CotG-E-G-F and CotG-E-G-R and CotG-C-G-F and CotG-C-G-R, respectively (shown in Table 1), and the CotG-E-G and CotG-C-G gene fragments were obtained after PCR amplification. The recombinant plasmids PDG1661-CotG-E-G and PDG1661-CotG-C-G were obtained by, respectively, connecting to *Bam*HI and *Eco*RI sites of the PDG1661 vector. Finally, the recombinant plasmids PDG1661-CotG-E-G and PDG1661-CotG-C-G were linearized, respectively, and transformed into BS168 through electroporation to obtain positive recombinant strains, which were named *B. subtilis* CotG-E-G and *B. subtilis* CotG-C-G.

**Table 1:** Primers used in this study.

Primers	Sequence (5′-3′)	Restriction enzyme site
CotG-E-G-F	CGGGATCCATGGGTCACTACTCTCACTCTGACA	*BamH I*
CotG-E-G-R	CGGAATTCTCATTTACCCCAGTTAGGTAAAC	*EcoR I*
CotG-C-G-F	CGGGATCCATGGGTCACTACTCTCACTCTGACA	*BamH I*
CotG-C-G-R	CGGAATTCTCATTTACGCAGTTAGGTCAA	*EcoR I*

Underlined nucleotides are restriction sites.

### Western blot

To detect the expression level of G protein, the recombinant *B. subtilis* CotG-E-G, CotG-C-G, and BS168 were cultured to the end logarithmic stage of bacterial growth. The bacterial solution was collected and washed three times with sterile PBS. Proteins were extracted from culture lysates by sonication. They were separated by 10% sodium dodecyl sulfate-polyacrylamide gel electrophoresis and then transferred to a nitrocellulose membrane. Anti-RABV G protein mAb (1:1,500) was used as the primary antibody and incubated at room temperature for 2 h. After that, HRP-conjugated goat anti-mouse IgG (1:15,000) was a secondary antibody. Electrochemiluminescence (ECL) Western Blotting Substrate was added, and the bands were captured using a Chemiluminescent Imaging System.

### Immunization and challenge

6-week-old female BALB/c mice were randomly divided into 4 groups, CotG-E-G and CotG-C-G experimental groups, BS168 negative control group, and PBS control group. Mice were orally administered 1 × 10^10^ spore/mouse with a stainless-steel round-tip gavage cannula at days 1–3, 14–16 and 28–30. The mice were monitored daily for clinical changes, including weight, hair, body temperature, eating habits and diarrhea.

Four weeks after the last immunization, mice were challenged intramuscularly with the RABV street strain HuNPB3 (100 × IMLD50). Observe and record changes in morbidity and body weight for 21 days. When the typical rabies symptoms appeared after the challenge, the mice were anesthetized with isoflurane and then humanely killed by cervical dislocation.

### Antibody detection

The mouse serum was collected at 0, 14, 28, 35 and 42 days after immunization, and the specific IgG antibody content in the serum was detected by enzyme-linked immunosorbent assay (ELISA). Antibody subtypes were detected 1 week after the last immunization. Briefly, inactivated and purified HuNPB3 was used as the coating antigen at a concentration of 1 μg/ml, loaded at 100 μl/well into 96-well microtiter plates and incubated overnight at 4°C. Incubate with 5% skim milk blocks for 2 h at 37°C. Serum to be tested was diluted 2-fold, 100 μl/well, and incubated at 37°C for 1.5 h. Then, HRP-conjugated goat anti-mouse IgG, IgG1, and IgG2a antibodies were added, at 100 μl/well, and incubated at 37°C for 1 h. Then add TMB substrate buffer to the culture dish, incubate in the dark for 10 min, stop color development with 2 M H_2_SO_4_, and read on OD450 microplate reader.

Fluorescent antibody virus neutralization (FAVN) (Cliquet et al., 1998) was used to detect the specific virus-neutralizing antibody (VNA) level in serum 1 week after the last immunization. Briefly, add the serum to be tested (4 replicates for each sample) in row 1, 50 μl/well, serially 3-fold serial dilutions to row 6 in a 96-well plate, and set up standard serum 0.5 IU/ml at the same time control. Then, 100 FFU of RABV CVS-11 virus was added to each well. Incubate in a 37°C 5% CO_2_ incubator for 1 h, add 2 × 10^4^ BHK cells to each well, and incubate at 37°C for 48 h, and then incubate with ice-cold 80% acetone and chamber for 30 min. and stained with FITC-labeled anti-RABV N protein antibody. The results were observed by a fluorescence microscope and calculated in IU/mL by comparison to reference serum.

### Detection of specific SIgA antibodies in feces

The level of SIgA antibody in feces was detected by the indirect ELISA. Inactivated and purified HuNPB3 was used as the coating antigen. The mouse feces collected every week were resuspended in pre-cooled PBS, and the supernatant was collected by centrifugation at 4°C and added to each well of the ELISA plate. Incubate at 37°C for 1.5 h, dilute HRP-conjugated goat anti-mouse IgA with 5% skim milk, 100 μl/well, and incubate at 37°C for 1 h. TMB substrate buffer was added to the plates for 10 min in the dark, and 2 M H_2_SO_4_ was added to stop the reaction, then read on a microplate reader at OD450.

### Splenocyte proliferation assay

One week after the last immunization, three mice in each group were euthanized, and their spleens were taken and crushed. The spleen cell suspension was filtered through a 70 μm filter, and the separated red blood cells were lysed with red blood cell lysis buffer. The splenocyte density was adjusted to 2.5 × 10 ^6^ cells/ml, and purified inactivated HNPB3 antigen (10 μg/ml) was seeded in a 96-well plate for 44 h, and then removed, CCK-8 was added at 10 μl/well and incubated for 4 h, place the 96-well cell plate in a microplate reader to read the OD450 nm value. The proliferation index (PI) was calculated as (OD stimulated culture–OD unstimulated culture)/(OD unstimulated culture–OD control culture).

### IFN-γ and IL-4 cytokine detection

Splenocytes were diluted to 2.5 × 10^6^ cells/ml, co-seeded with inactivated purified HNPB3 antigen (10 μg/ml) into 96-well plates, and incubated at 37°C, 5% CO_2_ for 24 h. IFN-γ and IL-4 secretion levels in splenocytes were detected using mouse enzyme-linked immunospot (ELISpot) kit. Finally, the spot-forming cells (SFCs) in each well were counted with an automated ELISpot reader.

### Statistical analysis

Statistical analysis was performed using GraphPad Prism 9.0 software, and results are expressed as mean ± SD. To determine the percent survival, Kaplan–Meier survival curves were analyzed using the log-rank test. Significance differences between groups were analyzed by one-way ANOVA or two-way ANOVA and were deemed significant at *p* values of 0.05 or less. Statistical significance is indicated as *p < 0.05, ** *p* < 0.01, ***p < 0.001, and ****p < 0.0001.

## Results

### Construction of recombinant *Bacillus subtilis*

**Figure 1:**
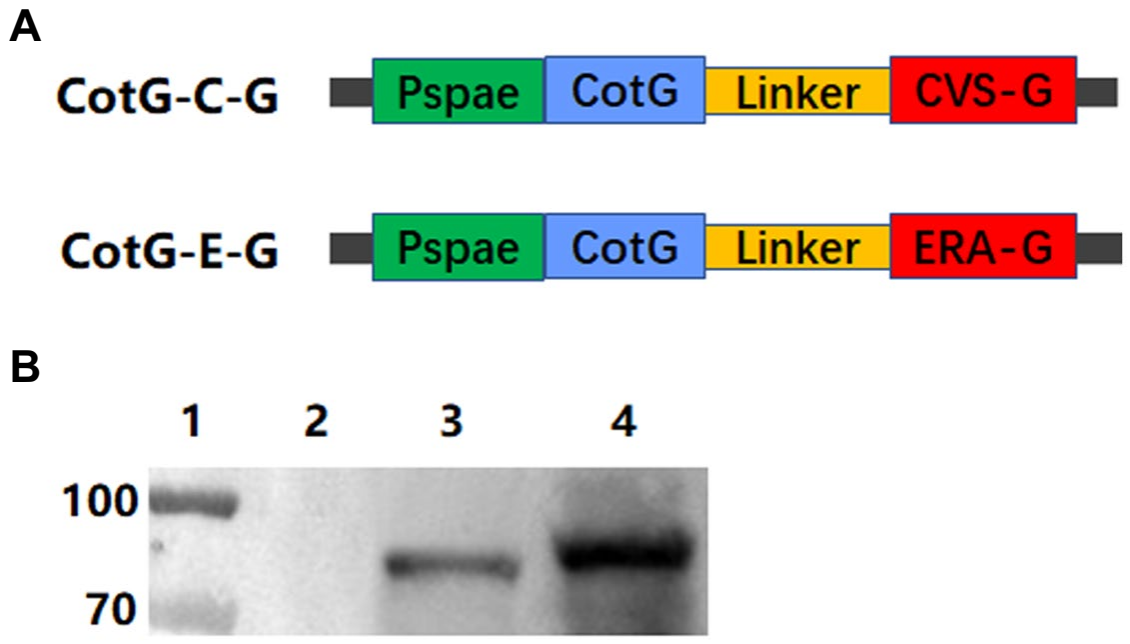
Construction and identification of recombinant *B. subtilis* expressing the immunogenic protein of RABV. **(A)** Schematic diagram of the construction of plasmids PDG1661-CotG-E-G and PDG1661-CotG-C-G expressing CotG-E-G fusion gene and CotG-C-G fusion gene, respectively. The Pspae represents promoter and the CotG represents spore capsid protein. **(B)** The expression of the G fusion protein in recombinant *B. subtilis* was detected by Western blotting. lane1: Maker; lane 2: *B. subtilis* BS168; lane 3: *B. subtilis* CotG-C-G; lane 4: *B. subtilis* CotG-E-G.

The construction strategies of recombinant *B. subtilis* CotG-E-G and CotG-C-G are shown in Figure 1A. The fusion genes CotG-E-G and CotG-C-G were, respectively, inserted into the vector PDG1661 with the promoter Pspae to obtain PDG1661-CotG-E-G and PDG1661-CotG-E-G, respectively. Then, the empty vectors PDG1661, PDG1661-CotG-E-G, and PDG1661-CotG-E-G were introduced into BS168 by electric shock transformation to obtain recombinant *B subtilis.* CotG-E-G and *B subtilis.* CotG-C-G. The lysates of *B. subtilis*. CotG-E-G and *B. subtilis.* CotG-C-G was identified by Western blotting. Two specific bands were detected with anti-RABV G protein mAb, whereas no bands were detected in the null strain (Figure 1B). The results showed that both recombinant *B. subtilis* were successfully expressed.

### Serum antibody detection

**Figure 2:**
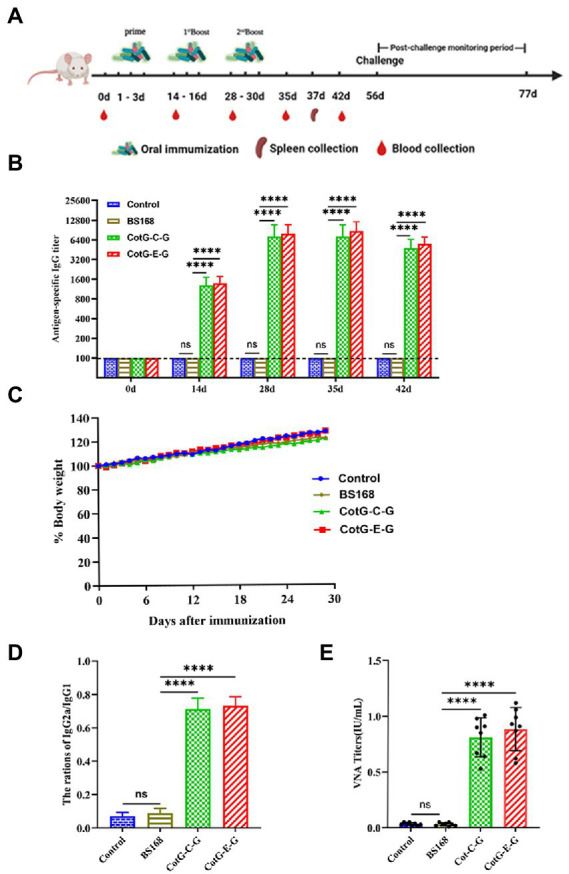
Specific anti-RABV antibody detection. **(A)** Immune scheme, BALB/c mice were randomly divided into 4 group, which were CotG-E-G, CotG-C-G, BS168 and PBS control group. Serum samples were collected at 0, 14, 28, 35 and 42 days (*n* = 8/ group). Spleens were collected from mice on day 37 (*n* = 3/group). **(B)** Changes of body weight in mice during immune period. **(C)** The levels of specific IgG (Serum dilution multiples start from 100 times, sequentially doubling dilution) antibodies in mouse serum were detected by indirect ELISA. **(D)** One week after the last immunization, the IgG2a/IgG1 ratio was determined by indirect ELISA. **(E)** One week after the last immunization, VNA titers were determined by the FAVN method. The mean and standard deviation of each group were analyzed using one-way or multi-way ANOVA (*p < 0.05, **p < 0.01, ***p < 0.001, **** *p* < 0.0001).

To evaluate the immunogenicity of the recombinant *B. subtilis* CotG-E-G and CotG-C-G, mice were orally immunized, and serum samples were collected after each immunization, the immunization program is shown in Figure 2A. No adverse effect, such as fatality, gloomy spirit, weight loss (Figure 2B) or diarrhea, was observed in mice during the whole immunization period. Serum-specific antibody levels were determined by indirect ELISA. The results showed that both CotG-E-G and CotG-C-G in the experimental group could detect obvious IgG antibody levels. On the 14th day after immunization, the antibody level of the experimental group was significantly increased, and the difference was extremely significant compared with the control group, on the 35th day after immunization, the serum IgG antibody levels of CotG-E-G and CotG-C-G in the experimental group reached the highest level (Figure 2C). These results indicated that recombinant *B. subtilis* CotG-E-G and CotG-C-G could effectively induce a systemic humoral immune response in mice.

The production of different antibody subtypes can reflect the type of immune response to a certain extent. To further understand the antibody responses induced by CotG-E-G and CotG-C-G, 1 week after the last immunization, IgG antibody subtypes were detected in the serum of mice 1 week after the last immunization. The results showed that the value of IgG2a/IgG1 was less than 1 (Figure 2D), indicating that after oral immunization, mice developed a Th2 immune response (biased to IgG1), which could increase the level of vaccine-induced humoral immune responses to a certain extent.

### Neutralizing antibody detection

To further determine whether recombinant *B. subtilis* CotG-E-G and CotG-C-G could induce the production of VNA, serum samples were obtained after the last immunization. No specific VNA was detected in the serum of mice in the control group, while the VNA titers of CotG-E-G and CotG-C-G in the experimental group were higher than 0.5 IU/ml, as shown in Figure 2E, according to the WHO that the RABV neutralizing antibody titer greater than 0.5 IU/ml has a protective effect on the body. The results showed that both recombinant *B. subtilis* have good immunogenicity.

### Detection of specific SIgA levels in feces

**Figure 3:**
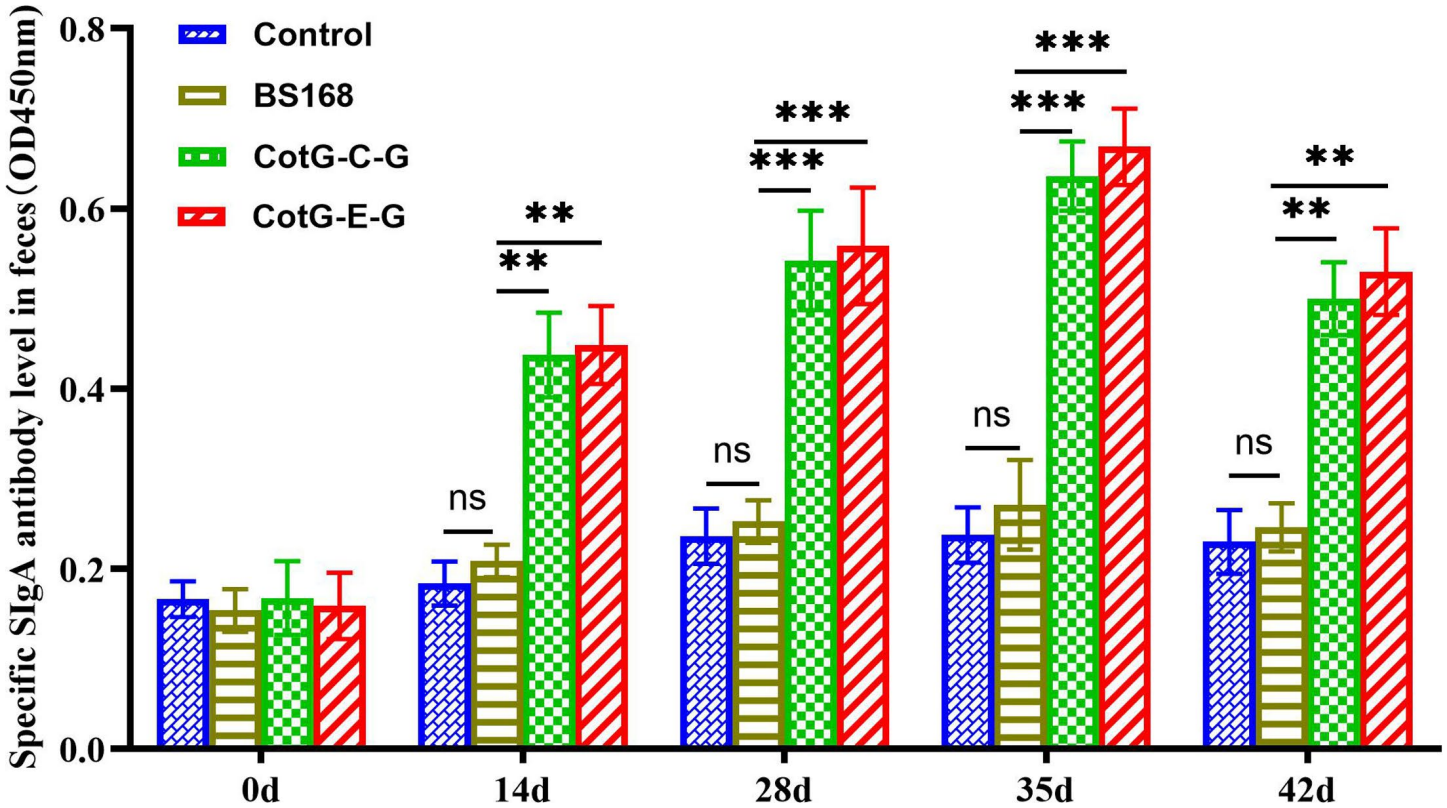
Detection of specific SIgA mucosal levels. The feces of mice (*n* = 8) were collected at 0, 14, 28, 35 and 42 days after the first vaccination, and the specific SIgA (the value of OD450 nm was calculated) level was detected by indirect ELISA. Data are presented as mean ± SD for each group (*p < 0.05, **p < 0.01, ***p < 0.001, ****p < 0.0001).

The specific SIgA antibody level was detected in the feces of mice after oral immunization. Significant SIgA antibody level could be detected in the feces of the experimental group from the 14th day after immunization, which is significantly higher than that of the control group, and the specific SIgA antibody level reaches the highest level on the 35th day after immunization (Figure 3), indicating that recombinant *B. subtilis* CotG-E-G and CotG-C-G could effectively induce mucosal immune response after oral immunization.

### Splenocyte proliferation assay

**Figure 4:**
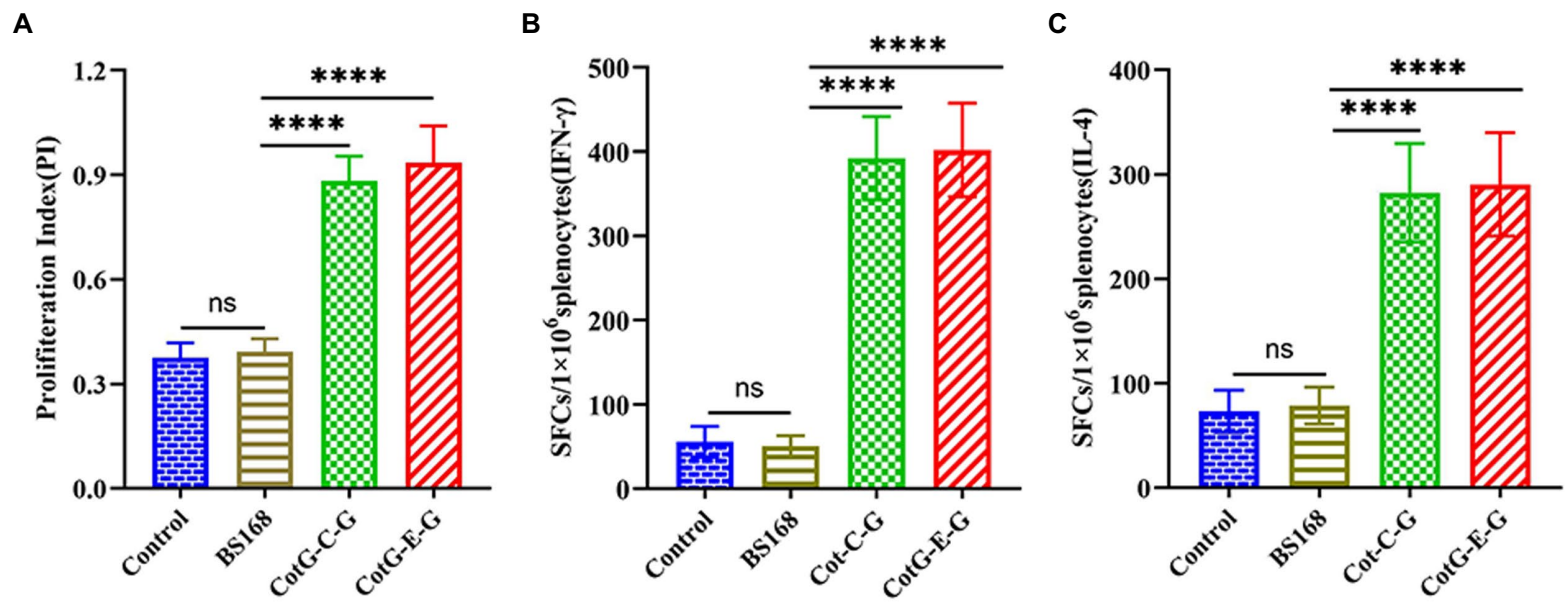
Splenocyte proliferation. On the 37th day after immunization, the spleens of mice were taken (*n* = 3/group), Splenocytes were stimulated with inactivated purified HNPB3 protein. **(A)** CCK-8 colorimetric assay for cell proliferation index. **(B,C)** The levels of IFN-γ and IL-4 secreted by splenocytes were measured by ELISpot method (The ordinate represents the number of spot-forming cells per well). Data are presented as mean ± SD of each group (*p < 0.05, **p < 0.01, *** *p* < 0.001, ****p < 0.0001).

To evaluate the effects of CotG-E-G and CotG-C-G on splenocyte proliferation in mice, *in vitro* splenocyte proliferation assay was performed 1 week after the last immunization. The splenocyte proliferation ability of CotG-E-G and CotG-C-G mice immunized with inactivated and purified HNPB3 protein was significantly higher than that of control mice (Figure 4A). The results showed that recombinant *B. subtilis* could promote the proliferation of immune cells and stimulate a strong antigen-specific immune response.

### Detection of splenic lymphocyte cytokines

To further investigate the antigen-specific cellular immune response, the levels of secreted IFN-γ and IL-4 in mouse splenocytes were detected by ELISpot assay. As shown in Figures 4B,C, the amounts of IFN-γ and IL-4 secreted in the splenocytes of CotG-E-G and CotG-C-G mice in the immunized group were significantly higher than those in the control groups, it is suggested that recombinant *B. subtilis* could enhance cytokine production, indicating that both Th1 and Th2 of acquired immunity were activated.

### Protection after the challenge of orally immunized mice

**Figure 5:**
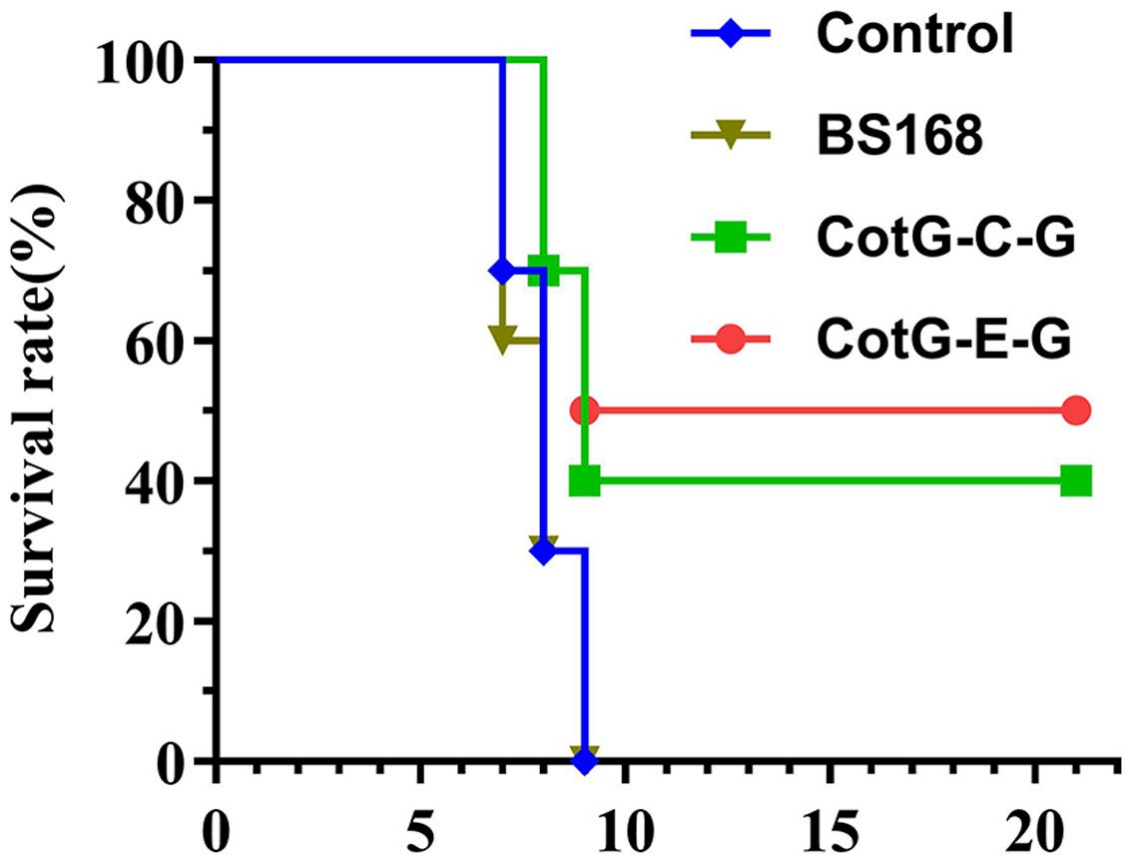
Protection of recombinant *B. subtilis* against lethal RABV challenge. Groups of mice (*n* = 10) were challenged with 100 × IMLD50 of RABV street strain 4 weeks after the last immunization. Observed for 21 days. The survival of mice in each group at different times after the challenge was recorded. The *p* value between BS168 and CotG-E-G group was 0.071, and BS168 and CotG-C-G group was 0.144.

To determine the protective efficacy of recombinant *B. subtilis* CotG-E-G and CotG-C-G. Four weeks after the last immunization, mice were challenged with a lethal dose of HNPB3 of 100 × IMLD50 and were monitored continuously for 21 days to observe their clinical symptoms and mortality. As shown in Figure 5, the control group mice all died within 10 days after the challenge. In contrast, individual mice in the experimental group CotG-E-G began to develop rabies symptoms such as convulsions and paralysis on the 8th day and were humanely sacrificed. After the 21-day observation period, the survival rate of CotG-E-G in the experimental group was 50%, and the survival rate of CotG-C-G was 40%. These results indicated that recombinant *B. subtilis* had good immunogenicity and a certain protective effect against lethal RABV attacks.

## Discussion

Stray animals and wild animals live in no fixed place and have a wide range of activities, so it is difficult to vaccinate against rabies through intramuscular injection. Therefore, the large-scale application of oral rabies vaccine (ORV) is the best way to solve the problem of rabies vaccination in stray animals and wild animals. The earliest oral immunization applied to wild animals is the attenuated live vaccine, mainly including ERA, SAD B19 and SAG-2 strains. ERA strain was the first to immunize red foxes as ORV in Europe, and achieved good immune effect (Baer et al., 1971; Steck et al., 1982). ERA strains were widely used to control fox rabies in North America between 1989 and 2009, greatly reducing the risk of human exposure to rabies (Rosatte et al., 2007, 2008). Wild animal ORVs prepared from attenuated rabies virus strains such as SAG2 and SAD B19 has been successfully used to control rabies in European foxes and raccoons in the United States and Canada, greatly reducing the spread of wild animal rabies (Wallace et al., 2020). Currently, the World Organization for Animal Health (WOAH) recommends two oral rabies vaccines, SAG2 and VR-G, for oral vaccination of dogs (Jackson, 2013). In 2021, Thailand will carry out the first large-scale application of ORV, and 83.0% (1,485/1789) of stray dogs will be immunized through ORV, significantly improving the vaccination coverage of stray dogs (Chanachai et al., 2021). Therefore, the oral rabies vaccine plays an important role in the global elimination of human rabies deaths from dog transmission.

Oral administration is simple and safe and has received more and more attention in vaccine development. In recent years, many oral rabies vaccines have been developed and evaluated, including recombinant live vector vaccines, attenuated vaccines, and recombinant virus vaccines. Given the safety and efficacy of the recombinant live vector vaccine, it can be used as a candidate vaccine for oral rabies vaccines. One of the current challenges in developing oral vaccines is that low pH in the gastric environment can destroy vaccine immunity. So, *B. subtilis* was selected as the oral vaccine delivery carrier in this study. *B. subtilis* has strong stress resistance, can resist the erosion of gastric acid in the gastrointestinal tract, and can effectively survive in the low-acid environment in the stomach and complex intestinal conditions (Zhou et al., 2008a,b; Li et al., 2022). *B. subtilis* exerts immune adjuvant activity and is effective against most oral antigens (Tanasienko et al., 2005). *B. subtilis* can germinate in intestinal antigen-presenting cells, where antigens can be processed and presented to downstream immune cells, inducing a strong mucosal immune response in the intestine (Duc le et al., 2004; Mauriello et al., 2007). In addition, the production cost of *B. subtilis* is low, the production process is simple, and it is easy to store, which reduces the cost of vaccine transportation. It is safe and non-toxic to humans and animals, and is certified as a GRAS product. Therefore, it can be used as an efficient delivery vehicle.

Isticato et al. (2001) first established a spore surface display system, using CotB as the anchor gene, the 459 amino acid fragment of the C-terminal of tetanus toxin (TTFC) was successfully displayed on the surface of *B. subtilis* by budding surface display technology. Using the fusion protein Cot B-TTFC of TTFC and CotB to immunize mice by oral and intranasal methods, the mice can produce mucosal IgA and systemic IgG immune responses. Kwon (Kwon et al., 2007) used the CotG gene of capsid protein as a fusion vector and successfully demonstrated galactosidase on the surface of *B. subtilis* bud hug. The recombinant bud hug had galactosidase catalytic activity in the water-organic reaction system. In addition, related studies have proved that *B. subtilis* probiotic strains did not cause significant adverse effects in acute toxicity tests (Yuan et al., 2012; Zhang et al., 2013), organs such as the heart and liver, indicating that *B. subtilis* is safe and non-toxic to mammals (Mingmongkolchai and Panbangred, 2018).

Serum-neutralizing antibodies reflect the neutralizing ability of the body to the virus, to a certain extent, it could replace the challenge study. RABV-neutralizing antibody assays are considered the gold standard for evaluating the rabies vaccine. The WHO and WOAH suggest that the RABV-VNA titer of 0.5 IU/ml has a protective effect on the body (Chen and Emergency, 2019). This standard has been applied in many reports (Tanisaro et al., 2010; Mittal, 2013). In this study, 1 week after the last immunization, the RABV VNA titers of CotG-E-G and CotG-C-G in the experimental group were higher than 0.5 IU/ml (Figure 2D). These results indicated that both recombinant *B. subtilis* strains had certain protective effects against RABV. Studies have shown that SIgA is the main antibody subtype and effector for body defense. SIgA antibody in intestinal mucosa has the function of neutralizing the virus, inhibiting virus invasion, and regulating the dynamic balance of mucosal surface (Liu et al., 2009; Kamada and Núñez, 2014). Serum immunoglobulin IgG antibody is an important indicator of the systemic immune response. In this study, after oral immunization of mice with recombinant *B. subtilis* CotG-E-G and CotG-C-G, the levels of RABV-specific SIgA in feces and IgG antibody in serum were detected. The results showed that the RABV specific SIgA and IgG antibody levels were significantly increased. These results indicated that the mice developed efficient humoral immune responses after oral immunization. Notably, specific antibodies induced by recombinant *B. subtilis* persisted for more than 35 days with high antibody levels, this may be related to the spores resisting digestion and germinating in the gut (Casula, 2002; Duc Le et al., 2004). In terms of cellular immune responses, the results showed that recombinant *B. subtilis* could induce spleen cell antigen-specific cell proliferation (Figure 4A), and effectively stimulate splenocytes to secrete IFN-γ and IL-4. IFN-γ is a Th1 cytokine involved in cellular immune responses, IFN-γ plays an antiviral role by promoting the lysis and clearance of virus-infected cells and inhibiting the expression and replication of viral genes (Venkataswamy et al., 2015). IL-4 is a Th2 cytokine associated with humoral immune responses, which drives the maturation of B cells into plasma cells, resulting in antibody production (Fang et al., 2007). This indicates that recombinant *B. subtilis* can effectively stimulate the production of Th1 and Th2 cytokines in mice, thereby enhancing cellular and humoral immune responses.

In conclusion, our results showed that immunization of mice with recombinant *B. subtilis* significantly increased the fecal-specific SIgA titers, serum specific IgG antibody levels, and serum neutralizing antibodies, it can induce the body to produce strong cellular and humoral immune responses, which is helpful to better resist the invasion of RABV. These data suggest that recombinant *B. subtilis* is expected to be a new and promising oral rabies vaccine candidate to prevent and control rabies in wild animals.

## Data availability statement

The datasets presented in this study can be found in online repositories. The names of the repository/repositories and accession number(s) can be found at: https://www.ncbi.nlm.nih.gov/genbank/, E-G: J02293. https://www.ncbi.nlm.nih.gov/genbank/, C-G: GQ918139.

## Ethics statement

The animal experiments were approved by the Animal Welfare and Ethics Committee of Changchun Veterinary Research Institute under the license number JSY-DW-2018-02. All BALB/c mice were treated according to the Guidelines for the Welfare and Ethics of Laboratory Animals of China (GB 14925–2001).

## Author contributions

YiZ and XX conceived and designed the experiments. YiZ, RM, SS, BL, and EL performed the experiments. YiZ and ZC analyzed the data. TW, YF, and SY contributed reagents, materials, and analysis tools. YiZ wrote the manuscript. XX, YoZ, and FY reviewed the manuscript. XX and YiZ requested financial support. All authors read and approved the final manuscript.

## Funding

This research was supported by the Jilin Provincial Science and Technology Development Program (20210202052NC).

## Conflict of interest

The authors declare that the research was conducted in the absence of any commercial or financial relationships that could be construed as a potential conflict of interest.

## Publisher’s note

All claims expressed in this article are solely those of the authors and do not necessarily represent those of their affiliated organizations, or those of the publisher, the editors and the reviewers. Any product that may be evaluated in this article, or claim that may be made by its manufacturer, is not guaranteed or endorsed by the publisher.
